# Intricate relationships between naked viruses and extracellular vesicles in the crosstalk between pathogen and host

**DOI:** 10.1007/s00281-018-0678-9

**Published:** 2018-05-22

**Authors:** Susanne G. van der Grein, Kyra A. Y. Defourny, Erik F. J. Slot, Esther N. M. Nolte-‘t Hoen

**Affiliations:** Department of Biochemistry & Cell Biology, Faculty of Veterinary Medicine, Utrecht University, Utrecht, The Netherlands

**Keywords:** Extracellular vesicles (EV), Exosomes, Infection, Naked virus, Antiviral immunity

## Abstract

It is a long-standing paradigm in the field of virology that naked viruses cause lysis of infected cells to release progeny virus. However, recent data indicate that naked virus types of the Picornaviridae and Hepeviridae families can also leave cells via an alternative route involving enclosure in fully host-derived lipid bilayers. The resulting particles resemble extracellular vesicles (EV), which are 50 nm–1 μm vesicles released by all cells. These EV contain lipids, proteins, and RNA, and generally serve as vehicles for intercellular communication in various (patho)physiological processes. EV can act as carriers of naked viruses and as invisibility cloaks to evade immune attacks. However, the exact combination of virions and host-derived molecules determines how these virus-containing EV affect spread of infection and/or triggering of antiviral immune responses. An underexposed aspect in this research area is that infected cells likely release multiple types of virus-induced and constitutively released EV with unique molecular composition and function. In this review, we identify virus-, cell-, and environment-specific factors that shape the EV population released by naked virus-infected cells. In addition, current findings on the formation and molecular composition of EV induced by different virus types will be compared and placed in the context of the widely proven heterogeneity of EV populations and biases caused by different EV isolation methodologies. Close interactions between the fields of EV biology and virology will help to further delineate the intricate relationship between EV and naked viruses and its relevance for viral life cycles and outcomes of viral infections.

## Introduction

Naked viruses are generally thought to leave cells by causing rupture of the infected cells, leading to the release of non-enveloped viral capsids containing the viral genome. However, several species of naked viruses have been described to exit infected cells before cell lysis occurs [1–7]. It has been long known that many of these viruses modify intracellular host membranes in order to form so-called replication organelles, which are specialized intracellular membranous structures where the viral genome is replicated [8]. Recent evidence shows that host membranes also play a crucial role in non-lytic virus release from intact host cells, by providing a membranous coat allowing the exit of naked viruses enclosed in small lipid bilayer-delineated vesicles [9]. These membrane-enclosed viruses, sometimes referred to as “quasi-enveloped viruses,” can cause effective infection of healthy cells [9, 10]. These findings aroused great interest because host membrane-enclosed naked virus particles can escape detection by the immune system and play an important role in virus propagation [11, 12].

The active release of membrane vesicles is not unique to virus-infected cells. A large body of evidence collected over the last 10–15 years indicates that virtually all cells release lipid bilayer-enclosed vesicles, collectively referred to as “extracellular vesicles” (EV). Release of such vesicles by cells has come in the limelight as a widespread and conserved means of intercellular communication (reviewed in [13]). Research in the EV field strongly progressed due to the development of specialized technology facilitating purification and in-depth characterization of different types of EV. Currently available data clearly indicate that cells can release heterogeneous populations of EV, which in part is caused by the existence of multiple EV biogenesis pathways. It has been hypothesized that EV-enclosed viruses arise due to convergence of EV formation pathways with those for virus assembly [11, 12]. In this review, the relationship between naked viruses and EV is discussed in light of what we know about diversity in EV biogenesis routes and the heterogeneous nature of EV. We stress the necessity to study the formation and function of EV-enclosed viruses in the context of the complete population of EV released by cells, which may change in composition depending on the time after infection and environmental conditions. The complexity of this research area pleads for strong liaisons between the fields of EV biology and virology to further unravel how virus-induced EV affect virus progression and disease.

## Ins and outs of extracellular vesicles

EV are lipid bilayer-enclosed vesicles with specific protein and nucleic acid cargo that are actively released by virtually all cell types (reviewed in [14]). Over recent years, it has become clear that EV can play an important role in various homeostatic and pathological processes by delivering specifically sorted cargo molecules to target cells (reviewed in [13]). EV can be formed by inward budding of late endosomal compartments, creating intraluminal vesicles (ILVs). When such multivesicular bodies (MVB) fuse with the plasma membrane, the ILVs are released into the extracellular environment and are then referred to as “exosomes.” Such exosomes are in general 50–150 nm in size. Alternatively, EV directly pinch off the plasma membrane. These vesicles are often referred to as “microvesicles” (MV), which vary in size from around 100 nm to a few micrometers. Proteins frequently detected in EV include tetraspanins such as CD9, CD63 and CD81, and endosomal proteins such as tumor susceptibility gene (TSG) 101 and syntenin (reviewed in [14]). In addition, EV contain cell type-specific proteins, such as MHC class II and specific integrins. Furthermore, EV carry several types of small non-coding RNAs, including miRNA, tRNA, and Y-RNA [15], which may regulate gene expression or induce signaling in target cells (reviewed in [16]).

Mechanisms underlying the formation and release of EV have been partly resolved (reviewed in [14]). Proteins of the endosomal sorting complexes required for transport (ESCRT) machinery are important for several steps in EV biogenesis. Vesicle formation can be initiated by binding of the ESCRT-0 protein HRS to cargo selected for incorporation into ILVs and to phosphatidylinositol 3-phosphate (PI3P) lipids. HRS recruits ESCRT-I proteins including TSG101, which subsequently recruit ESCRT-II. ESCRT-I and II play a role in the sorting and loading of ubiquitinated proteins within ILVs and the budding of these vesicles. ESCRT-III recruits the AAA ATPase VSP4 and thereby aids in the scission of budding vesicles. In addition, ESCRT-independent pathways exist in which ILV formation involves tetraspanin-enriched domains or conversion of sphingomyelin to ceramide by sphingomyelinases [17].

At the start of the field of EV research, the main focus was on small EV (50–150 nm), assuming that these represent true exosomes. More recently, it was demonstrated that viable cells also actively release larger types of functional EV [18, 19]. In addition, in-depth proteomic analysis suggests that populations of small EV contain both endosome-derived exosomes and non-exosomal EV [20]. These data indicate that cells simultaneously release various types of EV that differ in composition and function. Heterogeneity in EV populations is not only caused by variation in subcellular origin. The quantity and molecular composition of released EV is also highly influenced by the activation and differentiation status of the producer cell and external factors such as the availability of nutrients and oxygen levels [21, 22]. The variable cargo of released EV may be reflective of changes in the intracellular abundance of these molecules. However, specific cargo may also be selected for packaging in a highly regulated manner. As a result, EV can transfer protein, lipid or nucleic acid cargo molecules that inform distant or neighboring cells about the status quo of the producer cell.

The current lack of protein markers that non-circumstantially discriminate between one type of EV and the other has hampered the development of a practical EV nomenclature and comparison of data generated by different research groups. This is further complicated by the fact that a large variety of different EV isolation methods is being employed, each of which is biased in how efficiently specific EV subpopulations are isolated and to which extent the resulting EV populations are contaminated with co-isolated non-EV components. Hence, various initiatives were taken to standardize EV isolation and characterization methods and to improve correct reporting of experimental details, aiming to increase transparency and reproducibility of published results [16, 23].

## How do EV and viruses interrelate?

Early in the developing field of EV research, the small size of EV and their capacity to transfer genetic material to other cells already triggered thoughts on a potential relationship between EV and viruses [24]. Enveloped viruses, and retroviruses in particular, also resemble EV with regard to their enclosure in host-derived membrane, the molecular machinery driving their formation in the endosomal system or at the plasma membrane, and strategies for uptake by target cells [25, 26]. This analogy fed the idea that viruses could have evolved to usurp EV-mediated communication for the delivery of viral products and/or host factors beneficial to the virus to neighboring cells. The role of EV in the transfer of viral subunits has been extensively studied and reviewed for many enveloped viruses, but in particular for HIV and EBV [12, 26–28]. Upon infection, these viruses induce the release of EV containing viral products that function in the conditioning of the surrounding environment to enable optimal virus propagation. Conversely, some of the EV-associated molecules can activate the innate antiviral immune response, substantiating that such EV can also have pro-host effects. Although there is strong interest in further delineating the role of EV in retroviral infections, technical limitations exist in the efficiency with which EVs that carry viral proteins, host proteins, and viral genomic elements can be separated from enveloped viral particles that carry similar molecules [26].

With regard to naked viruses, the interplay with EV is illustrated by the reported packaging of complete virions in host membrane-derived vesicles containing several transmembrane and luminal proteins characteristic for EV. This has been primarily shown for viruses belonging to the Picornaviridae family, which includes the causative agents of a large list of human and animal diseases. Electron microscopy (EM)-based studies demonstrated EV enclosure of hepatitis A virus (HAV), coxsackievirus serotype B3 (CVB3), and poliovirus (PV) [9, 29, 30]. Host membrane enclosure of virus particles was also shown for unrelated naked viruses such as hepatitis E virus (HEV) [31] and suggested for rotavirus (Reoviridae) [4]. Association with EV offers naked viruses the opportunity to escape cells in an early stage of infection and to hide from the immune system. Current research in this field focuses on unraveling how pathways for virus release and EV biogenesis converge and on the functional analysis of EV-enclosed naked viruses (hereafter referred to as “EV-virus”). Until now, EV-virus-related studies have been performed using several types of naked viruses with different replication kinetics. This inevitably implies that there is a high level of variability in experimental conditions under which EV-virus formation is studied. Although this may reduce inter-study comparability of data, it also stimulates hypothesis formation on conservation of EV-virus biogenesis pathways among (groups of) viruses. Below, we aim to identify virus-related and environmental factors that can influence EV-virus formation, release, and function.

## Overlap in mechanisms for EV-mediated release between different naked virus types?

EV membranes surrounding naked virus particles are host-derived because these viruses do not encode any structural membrane proteins. However, the subcellular origin of the host membrane surrounding the viral particles is poorly defined and their routes of formation remain largely elusive. Moreover, it is questionable whether infection by different naked virus types induces EV-mediated release of virus particles via similar pathways. In most of the reported studies, (limited) characterization of the EV-virus-associated host proteins, in particular those known to be involved in EV formation, has been performed (Table 1). Based on these datasets, two routes of EV-virus formation have been suggested, in which either autophagosomes or late endosomes are regarded as sites where membrane enclosure of virus particles occurs.

**Table 1:** Protein composition of EV-virus in relation to EV isolation methods

Virus		EV-virus protein composition	Method of detection	Pre-clearing	EV isolation	Ref
Enterovirus (Picornaviridae)	PV	LC3-II Calnexin	WB	500×*g* 5 min	Bead capture (AnnV) (on 5,000×*g* 10 min pellet)	[9]
	CVB3	Flotillin-1 LC3-II	WB	3,000×*g* 15 min	Commercial reagent-based precipitation	[30]
		CD63 LC3 TOM70 DRP1	WB	3,000×*g* 15 min	Commercial reagent-based precipitation	[32]
	EV71	CD63	WB	300×*g* 20 min 2,000×*g* 20 min 10,000×*g* 30 min	100kD ultrafiltration, UC: 100,000×*g* 30 min through sucrose	[10]
		CD81 LC3	WB	n.s.	Commercial reagent-based precipitation	[33]
Hepatovirus (Picornaviridae)	HAV	ALIX Flotillin-1	WB	1000×*g* 10 min 2× 10,000×*g* 30 min	UC: 100,000×*g* 60 min, density gradient (EV at 1.06–1.10 g/ml)	[29]
		ALIX CD9 DPP4 CHMP4 CHMP7 VPS4B	Proteomics and bead-based detection	2 × 10,000×*g* 30 min	UC: 100,000×*g* (time n.s.), density gradient (EV at 1.02–1.08 g/ml)	[34]
		LAMP1 RAB7A RAB5C Syntenin	Proteomics			
Orthohepevirus (Hepeviridae)	HEV	CD81	WB	1,200×*g* 10 min 10,000×*g* 30 min	UC: 100,000×*g* 60 min	[31]
		CD9 CD63 CD81	Bead-based detection	Filtration 0.22 μm	UC: 100,000×*g* 70 min Surface protein-specific capture (CD9/CD63/CD81)	[35]
		ALIX CD63 TSG101	WB	300×*g* 15 min 2,000×*g* 20 min 10,000×*g* 30 min	UC: 110,000×*g* 70 min density gradient (EV at 1.10–1.15 g/ml)	[36]
		TGNOL2	Bead-based detection	–	Surface protein-specific capture (TGNOL2) in culture supernatant	[37]

Listed are studies describing the protein composition of virion-containing EV derived from picornavirus-infected cells (enterovirus and hepatovirus genera), as well as HEV-infected cells (Hepeviridae). Indicated proteins were detected by western blot analysis, proteomics, or bead-based detection of proteins co-captured with viral RNA. For proteomics studies, only proteins relevant to EV biogenesis pathways are listed. Clean-up steps performed prior to EV-virus isolation included centrifugation at various g-force or filtration. EV-virus isolation methods also varied between studies and included particle sedimentation-based techniques (commercial reagent-based precipitation and ultracentrifugation), isopycnic density gradient centrifugation and capture on affinity purification beads. n.s. = not specified, WB = western blot, UC = ultracentrifugation

### An autophagy-related route of EV-virus formation

Several enteroviruses, such as CVB3, PV, and EV71, were reported to escape cells enwrapped in EV containing not only well-known EV marker proteins like CD63, CD81, and flotillin-1 [30, 32, 33], but also the autophagy-related protein LC3 [9, 30, 32, 33]. This finding triggered the idea that autophagy could be involved in formation of EV-virus. Autophagy is a cellular catabolic process by which cells under stress conditions engulf and break down damaged or unnecessary cytoplasmic constituents and recycle the building blocks to fuel processes that are crucial to survival. It is well established that autophagy plays an important role in virus infection. Many different picornaviruses, including PV, rhinovirus, EV71, CVB3 (genus enterovirus), and FMDV (genus aphthovirus) actively induce autophagy to sustain infection [38–42]. During autophagy, LC3 is involved in selective cargo sequestration, as well as elongation and closure of the double membrane phagophore to form autophagosomal compartments (reviewed in [43]). Unlike other components of the autophagy regulatory machinery, LC3 in its lipidated form (LC3-II) decorates both the inner and outer membrane of autophagosomes. The reported presence of LC3 on EV containing naked virus particles suggests that autophagosomes may provide the membrane for EV-virus formation. In support of this idea, it was shown that disruption or stimulation of autophagy initiation appeared to respectively inhibit or boost the non-lytic spread of PV, without affecting virus replication [1]. These data support the idea that autophagy plays a multifaceted role in picornavirus replication and release. Interestingly, CVB3 infection was shown to not only initiate autophagy but also to block the autophagy flux towards lysosomal degradation [9, 44]. This suggests that virion-containing autophagosomes may not follow conventional routing towards lysosomes, but rather fuse with the plasma membrane to expel their contents to the external milieu. This route, coined “secretory autophagy” (reviewed in [45]), is an alternative disposal pathway for aggregated, defective, or non-functional cytoplasmic constituents to alleviate stress caused by these products under conditions of lysosomal dysfunction. Via this secretory autophagy pathway, membrane-bound vesicles decorated with LC3 could be released, provided that the autophagosomal inner membrane is not degraded. EV release via the secretory autophagy pathway has also been described in various non-infectious conditions. In neurodegenerative disorders, for example, this pathway was shown to drive the release of α-synuclein and other prion-like proteins in EV that display LC3 and tetraspanins CD9, CD63, and CD81 [46–51]. A role for secretory autophagy is also recognized in controlling the EV-mediated release of members of the IL-1 family of cytokines, including IL-1β and IL-18, which lack an N-terminal signal peptide needed to enter conventional protein secretion pathways [52, 53]. To further delineate the relationship between autophagy and EV-virus release, it is important to know whether and how picornaviruses that are proposedly released in LC3-decorated EV actively steer the autophagy pathway towards a secretory rather than degradative process. In this light, it is interesting to note that syntaxin 17, a factor required for fusion of autophagosomes with lysosomes, was sequestered away from autophagosome-like organelles that contained virions in CVB3-infected cells [9, 54].

### MVB-mediated release of hepatotropic naked viruses in EV

In contrast to the group of viruses described above, EV enclosing the hepatotropic viruses HAV (Picornaviridae) and HEV (Hepeviridae) reportedly did not contain LC3 and their release was not affected by inhibition of autophagy [29, 34]. Based on their protein composition, these EV-virus particles were instead proposed to form through inward budding into endolysosomal compartments and to be released upon fusion of MVBs with the plasma membrane (reviewed in [55]). EV containing HAV or HEV were strongly enriched for components of the endolysosomal pathway and regulators of vesicular transport, such as flotillin-1, syntenin, the Rab GTPases RAB5C and RAB7A and the tetraspanins CD9, CD63 and CD81 [29, 31, 34, 35]. In addition, EV-containing HAV and HEV comprised many ESCRT components and their accessory proteins [29, 34, 36]. The involvement of several of these host proteins, e.g., VPS4A and VPS4B, in the biogenesis of EV-enclosed HAV or HEV was confirmed by RNA interference (RNAi) studies [29, 34, 56]. However, the pathways of their formation do not entirely overlap. HAV-containing EV were shown to be highly enriched for ESCRT-III-associated proteins like ALIX and depletion of this protein abrogated extracellular release of HAV [29, 34]. HRS (ESCRT-0) and TSG101 (ESCRT-I), on the contrary, were neither incorporated in HAV-containing EV nor affected their release [29, 34]. Since ALIX was shown to directly interact with HAV capsid protein, a model was proposed where this interaction orchestrates the sorting of HAV virions for budding into MVBs while bypassing the need for other ESCRT proteins [29, 34]. Other than for HAV, EV-mediated release of HEV required the ESCRT-0/I proteins HRS and TSG101 [31, 56]. In addition, the non-structural viral protein ORF3, which can interact with TSG101, was shown to mediate extracellular release of HEV [56, 57]. Similarly, a non-structural protein of Bluetongue virus (Reoviridae) was also shown to connect the viral capsid to TSG101 [2, 58]. Thus, although EV-virus containing HAV and HEV both appear to originate from MVB, each of these viruses has evolved a unique mechanism to gain access to the MVB-mediated EV secretory route. Overall, enteroviruses and hepatotropic viruses can hijack several different host cellular processes involved in shuttling of cytoplasmic cargo into EV. However, since the MVB and secretory autophagy pathways do not operate autonomously and share many regulatory factors, resolving EV-virus formation pathways based on their molecular composition is challenging.

### Experimental conditions and EV-virus data interpretation

**Fig. 1:**
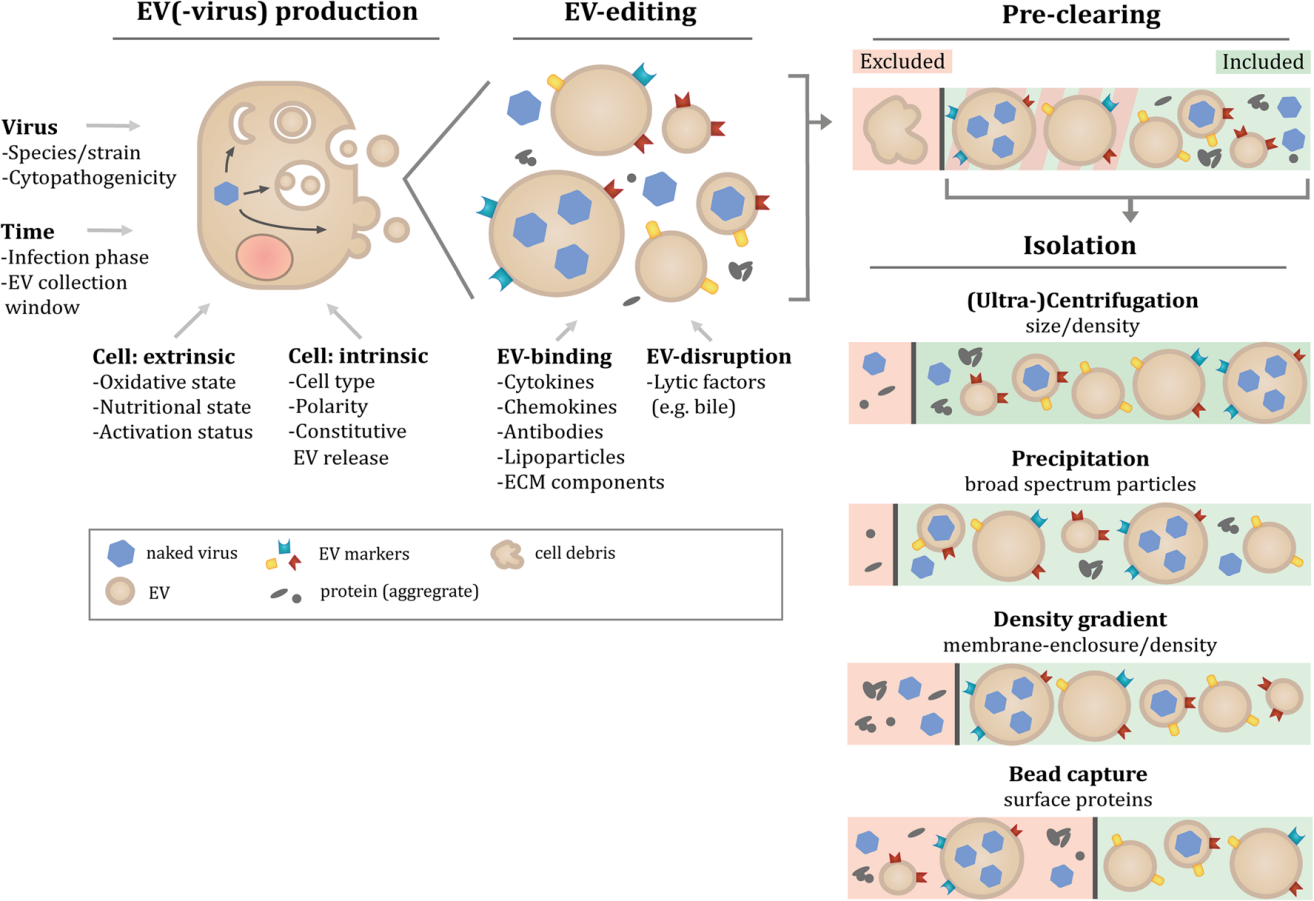
Multiple factors can influence the composition of EV-virus isolates. The figure presents a schematic overview of factors identified in the EV- and EV-virus-fields that affect the molecular composition of EV isolates. First, *EV*(*-virus*) *production* itself can vary based on factors relating to the producing cell, including the nature of the cell (intrinsic factors) and its environmentally determined condition (extrinsic factors). Upon infection, these factors coalesce with the properties of the virus in a *time*-sensitive manner to govern the production and release of virions, EV-virus, and other EV by the infected cell. Secondly, the heterogeneous population of released EV can undergo '*EV-editing'* by engaging with factors encountered in the extracellular environment. These factors can either bind to or disrupt EV membranes to modify the existing particles. Additional variation in the composition of EV isolates is introduced during *isolation and purification steps*. Depending on the centrifugal force applied in pre-clearing steps to remove cell debris, subsets of larger EV may either be depleted in this step or may be co-isolated in subsequent steps. The different techniques applied to isolate EV-virus are based on different principles related to physical, affinity or biochemical characteristics of EV. These EV isolation techniques therefore differ in the efficiency with which EV can be separated from contaminating naked virions and protein aggregates, or may specifically enrich for certain EV subtypes

A large body of evidence acquired in the EV research field has indicated that pre-analytic variables and the applied methodology to isolate EV have a major impact on both the quantity and type of isolated EV and co-isolated contaminants (reviewed in [59, 60]). The interpretation of currently available data on the molecular composition of EV-virus (Table 1) should therefore be evaluated in the context of experimental variation between studies. In the EV-virus studies described in this review, a large variety of fast- and slow-replicating virus strains and more or less susceptible cell lines was used. Besides cell culture- and virus-related variables (Fig. 1), also differences in the applied EV isolation methodologies compromise comparability of data obtained in different EV-virus studies. EV isolation techniques differ in the efficiency with which EV subpopulations can be isolated and separated from contaminating particles. Frequent contaminants of EV preparations include extracellular structures that overlap with EV in terms of size and density, such as protein aggregates or lipoprotein particles [61, 62]. The research strategies applied in EV-virus studies show large differences in both the stringency by which EV-containing culture supernatants were pre-cleared of contaminating cell debris and in EV isolation methods (Table 1). In some studies, all material pelleting at a centrifugal force up to 10,000×*g* was discarded in the pre-clearing step, while it is increasingly recognized that larger EV (often termed microvesicles) sediment at this speed. Such larger EV were shown to be phenotypically and functionally different from small EV sedimenting at 100,000×*g* [63–65]. In other studies, these larger EV were co-isolated with smaller EV because pre-clearing steps were performed at lower centrifugal force. Following pre-clearing, the types of EV isolation methods employed in the EV-virus studies included sedimentation of EV by either precipitation-based techniques or high-speed ultracentrifugation (Table 1). While high-speed ultracentrifugation may lead to sedimentation of a more restricted set of particle types, both techniques co-isolate protein and lipoprotein complexes [66]. In some studies, EV-virus was further purified by either density gradient ultracentrifugation, which separates EV from contaminating protein aggregates ([66, 67], or by affinity capture onto beads. Capturing moieties coated on these beads included antibodies to the common EV-associated proteins CD9, CD63, and CD81 for capturing EV-enclosed HAV or HEV [34, 35] and the phosphatidyl serine (PS) binding protein annexin V for capturing EV-enclosed PV [9, 68]. Although the risk of co-isolating contaminants is low, this technique is biased towards isolating only a subset of EV with the highest affinity for the beads [69–71] and will therefore only provide information on a particular subset of the total EV population. Taken together, different EV isolation and characterization techniques may specifically enrich for certain EV subtypes or fail to deplete contaminants (Fig. 1). This highlights the need for caution when drawing conclusions about the origin and biogenesis pathway of EV-virus based on the molecular composition of EV isolates.

## EV-enclosure affects the spread of naked viruses and antiviral responses

Enclosure of naked viruses in EV can influence several steps in both virus spread and the induction of or escape from antiviral responses. Most obviously, release via EV offers naked viruses an alternative egress route without compromising host cell integrity [9, 72] and protection from circulating neutralizing antibodies [10, 29, 35, 36, 73]. However, the EV membrane coat and co-transfer of host signaling molecules via EV can influence several other steps in virus dissemination, as the molecular composition of EV is a key determinant of the specificity and efficiency with which EV bind target cells as well as the functional response they elicit.

### EV-virus and naked virus particles differ in biodistribution

Upon release, EV generally bind to designated target cells that may be present in the local environment as well as at distant sites to exert their functional effects [74]. A similar process is seen during infection, where virus spreads locally or undergoes (systemic) dissemination. However, enclosure in EV can lead to changes in the spread of virions to different tissues. EV carrying EV71 infectivity, for example, preferentially spread infection to the liver and spleen (sites known to accumulate EV [75]), and less to the brain and intestine when compared to naked virus particles [76]. Similar to spreading of other types of EV [75, 77], the tissue-targeting of EV-virus may depend on the site of release, the parental cell type, and the molecular composition of the EV, features that likely differ per type of virus infection [75, 77]. In addition, differences in physical properties between EV and naked viruses, such as size, could contribute to the variation in the dissemination patterns among these two types of particles.

### Host membrane constituents regulate EV-virus attachment to cells

A wide range of receptor-ligand interactions have been identified that underlie interactions between various types of EV and their target cells. These include protein-protein (e.g., integrins), protein-sugar (e.g., lectins), and protein-lipid (e.g., PS lipid) interactions, the combination of which can be considered as an “address label” to direct EV delivery (reviewed in [25, 78]). A remarkable example of such interactions mediating the specific targeting of EV is the organotropism of tumor-derived EV, of which the tissue localization was shown to depend on particular integrins present on the EV surface [77]. Moreover, we previously showed that T cells depended on activation-induced conformational changes in the LFA-1 integrin to bind DC-derived EV via ICAM-1-LFA-1 interactions [79]. Our current understanding of the molecules that mediate uptake of EV-virus, and how this varies with the type of virus and infected cell type, is still limited. So far, EV surface exposure of PS lipid, a motif previously implicated in the uptake of EV into a variety of cells [80–83], was shown to be required for the uptake of EV-enclosed PV and HAV particles [9, 84]. In fact, the PS-receptor TIM-1 is currently the only confirmed receptor contributing to the uptake of EV-virus, as it was shown to promote adherence to and initial infection of kidney epithelial cells by EV-enclosed but not naked HAV virus particles [85]. Data regarding the role of TIM-1 in HAV infection for other cell types, however, is conflicting [29, 85]. Besides binding specificity, EV membranes surrounding naked virus particles can also affect the kinetics of target cell binding and subsequent infection efficiency. EV containing HEV or HAV, for example, bound their respective target cells with lower efficiency compared to naked virions, and hence displayed delayed infection kinetics [85, 86]. EV-enclosure of PV, on the other hand, enhanced the infection efficiency of macrophages, cells known to selectively take up PS-rich EV [9, 82, 83].

Interestingly, EV-enclosure can also allow some viruses to gain access to cells that are non-susceptible or non-permissive to their naked counterparts, which was shown to either boost the antiviral immune response or enhance virus spread. EV-enclosed HAV particles, for instance, could be effectively internalized by non-permissive plasmacytoid dendritic cells (pDC), thereby inducing interferon (IFN)-α production and promoting the antiviral immune response [84]. In contrast, EV carrying EV71 infectivity enhanced virus spread by promoting infection of an otherwise non-susceptible cell line lacking the EV71 entry and uncoating receptor SCARB2 [76, 87]. This finding possibly concurs with the idea that EV carrying viral RNA in the absence of a capsid can lead to infection of recipient cells in a receptor-independent manner [76]. Overall, however, our current knowledge on whether and how EV-virus affects viral tropism is limited.

### EV-mediated delivery of virus and host components to recipient cells

After engaging with a recipient cell, molecules in the lumen of EV can only exert functional effects upon liberation from the EV particle. Data demonstrating functional delivery of EV-enclosed RNA molecules gave rise to the assumption that fusion between EV and host cell membranes induces the direct release of EV cargo into the target cell cytoplasm. Such fusion events have been proposed to take place at the plasma membrane or in endosomal compartments upon internalization via multiple endocytic pathways, yet evidence demonstrating their occurrence is still scarce [78, 88–90]. In contrast, studies on EV-virus suggest that the EV content is liberated in the lumen of acidic endosomal/lysosomal compartments. This notion was based on the finding that infection of target cells by EV-enclosed HAV and PV particles was inhibited by blocking the interaction between the viral capsids and their respective cellular receptors, an interaction that can only occur after escape from the EV membrane [9, 29]. Moreover, EV-enclosed HAV and HEV particles were shown to require endosomal acidification to mediate infection, whereas this was not the case for naked viruses [29, 86]. These data indicate a role for EV degradation in late endosomes or lysosomes to release enclosed virus particles, a hypothesis that is supported by the finding that lysosomal factors involved in lipid/membrane degradation were required for EV-mediated infection of HEV [86]. Upon release, the interaction between virus particles and cellular receptors likely enables virion uncoating and release of the viral genome in order to establish a productive infection. Noticeably, endosomal/lysosomal release of EV-enclosed virions not only enables infection, but also poses a risk for virus degradation and immune activation. Internalization of EV-enclosed HAV by pDC, for example, was shown to boost the release of IFN-α by endosomal Toll-like receptor 7 (TLR7) recognition of viral RNA [84]. Similarly, protein components of degraded virions could serve in the activation of the adaptive immune response, as EV have been demonstrated before to be capable of providing antigens for MHC class II loading and antigen presentation by DC [81, 91].

The multicomponent nature of EV-virus inevitably causes a variety of bioactive molecules of host and/or virus origin to be co-delivered to the same target cell. Importantly, this allows these molecules to act in a cooperative manner to modulate recipient cell behavior and virus spread. EV71 infection, for example, was shown to selectively drive EV incorporation of both viral RNA and host cell-derived miRNA-146a, a negative regulator of innate immune activation and an inhibitor of IFN-α/β. In line with its function, the presence of this miRNA promoted EV-mediated infection of new cells in vitro and in vivo [76]*.* In addition, EV can simultaneously deliver multiple enclosed virus particles [9, 29, 30, 92]. This was postulated to facilitate genetic cooperativity, where individual virus copies that differ in mutational load can share viral protein machineries to facilitate successful infection. As a result, virus particles with an otherwise decreased fitness could escape potential innate immune recognition [11].

### EV-virus release and function in vivo

To understand the in vivo role of EV in general and EV-virus in particular, characterization of EV in body fluids of patients and animal models is being employed with increasing frequency to validate and guide in vitro studies [29, 72, 76, 85, 93–95]. Initial studies on EV-enclosed HAV and HEV particles in vivo revealed the predominant presence of EV-enclosed virus in serum samples, whereas feces contained mostly naked virions [29, 72, 93]. This stresses the importance of evaluating multiple types of patient samples for the presence of EV-virus. Moreover, in vivo EV-virus studies are complicated by the fact that mixtures of infected and non-infected cells, as well as permissive and non-permissive cells, can engage in reciprocal signaling cascades. The virus-induced cytokine IFN-α, for example, was shown to increase expression of interferon-stimulated genes (ISGs), such as APOBEC3G and IFITM3, in uninfected cells. These proteins can be sorted into EV along with mRNAs encoding ISGs and antiviral miRNAs [96–98]. EV containing these antiviral molecules protected recipient cells against infection, thereby constituting an antiviral strategy that likely bypasses the inhibition of type-I IFN response by viruses in infected cells [96–98]. In concordance, inhibition of EV release in vivo was shown to diminish IFN-α antiviral activity [97]. These data indicate that parallel to the induction of EV-virus release by infected cells, surrounding cells may release subsets of functionally different EV in response to virus infection. As a result, the heterogeneity among the EV induced upon infection in vivo is likely far more complex than observed in vitro*.*

## Connecting the state of knowledge on EV to future EV-virus studies

Over the years, researchers in the EV field unraveled several morphological, compositional and functional aspects of EV released by several cell types in healthy and pathological conditions. In addition, a large amount of technical knowledge has been acquired on the possibilities and limitations of a wide range of methodologies used to study EV. This knowledge should be optimally used to design future EV-virus studies. To illustrate this, three perspectives are described below.

### Naked virus-infected cells likely release multiple types of EV

Knowledge from the EV field indicates that cells can release various types of EV, which are heterogeneous with regard to size and molecular composition [20]. It is therefore expected that naked virus-infected cells not only release virus-containing but also other types of EV. Data substantiating this idea were recently published by McKnight et al., who showed that all HAV-containing EV carry the apical membrane protein DPP4 (dipeptidyl peptidase 4), but that only some of these EV carry CD9, CD63 and CD81 [34]. These results illustrate that virus-infected cells, like cells in non-infectious settings [20], release heterogeneous populations of EV. Mixed populations of EV not only arise due to differences in their subcellular origin, but also due to cell polarity. EV released from apical and basolateral sides of non-infected epithelial cells, for example, differ in molecular composition and function (reviewed in [99]). Similarly, HAV-containing EV were shown to be released from both the apical and basolateral side of epithelial cells [72], but it is yet unknown how these EV-virus subsets may differ in molecular composition and function. In addition, cell culture conditions can contribute to heterogeneity in released EV. The omission of serum in cell culture medium of some EV-virus studies [9, 31], for example, may have triggered starvation-induced EV release [100, 101] on top of virus-induced release. Given that naked virus particles can also be released from intact cells [9, 29, 72], awareness needs to be raised that the extracellular space between naked virus-infected cells contains a highly complex mixture of particles (Fig. 1). Many of the currently used EV characterization methods focus on identification of molecular components in bulk isolates of EV, and are therefore not well-suited to address EV heterogeneity. Recently developed high-throughput single EV detection methods, such as flow cytometry-based techniques and ImageStream analysis [102–104], can aid in determining which molecular components truly associate with EV-virus.

### Time is an important factor in studying virus-induced EV release

EV are often regarded as snapshots of cells. By definition, a snapshot represents the state of a system at a particular point in time. The type and number of released EV is indeed affected by various factors influencing the cell state (Fig. 1), including cell-intrinsic factors (e.g., cell type, tumorigenic state) and external factors (e.g., growth factors, microbial agents, oxygen level, type and level of nutrients). However, the speed with which signals imposed on cells translate into changes in EV release is variable. We previously showed that IgE-mediated stimulation of mast cells induced massive release of particular subsets of EV as rapid as 1.5 h after stimulation [105], whereas effects of LPS stimulation on EV release by dendritic cells become apparent over longer periods of time [102, 106]. Hence, timing should be considered as an important factor in relating the quantity and composition of EV to the activation or differentiation status of cells.

Temporal changes in EV composition also add an additional layer of complexity to the heterogeneity of virus-induced EV populations (Fig. 1). During virus infection, the state of cells rapidly changes while cells undergo a dramatic shift from homeostasis to pathology-related imbalances in a variety of cellular processes. The speed with which these changes occur in in vitro cell systems is highly dependent on the type of (laboratory-adapted) viral strain, host cell type, and infection conditions. Accurate assessment of temporal changes in host and viral molecules during the course of infection, e.g., by performing “quantitative temporal viromics” [107], could in the future reveal indicator molecules hallmarking the timing of the infection process. Relating the expression of such molecules to the type and number of released EV may be used as a strategy to increase comparability of data and to more reliably assess how EV release relates to cell status during virus infection.

### Tissue-specific EV-editing events can modify the function of virus-containing EV

Besides environmental factors that affect EV composition by modifying the parental cell status, components present in the extracellular milieu of tissues or in body fluids can additionally affect the composition and integrity of EV after their release. Such ‘EV-editing’ events (Fig. 1) include the binding of antibodies, coagulation factors or lipoparticles to the surface of EV [62] (reviewed in [108, 109]), and degradation of the EV membrane by lytic factors. Several lines of evidence indicate that this type of modifications can strongly affect the function of EV. Autoantigen-containing EV in synovial fluid of rheumatoid arthritis patients, for example, were shown to bind anti-citrullinated peptide antibodies, thereby forming immune complexes that induced strong pro-inflammatory responses [110]. Moreover, neuron-derived EV bind Alzheimer disease-associated amyloid β-peptide, thereby enhancing the formation of non-toxic amyloid fibrils and uptake by microglia [111]. Additionally, both EV and retroviruses were shown to bind to the extracellular matrix protein fibronectin, which occurs in soluble form in plasma, leading to enhanced target cell binding of EV and modulation of virus infectivity [112–115]. For the EV-enclosed naked viruses, multiple studies indicate that tissue-specific factors affect the integrity of the EV-virus membrane. The observations that HAV and HEV appear in feces as naked viruses, whereas in blood they are enclosed by EV [29, 72, 93, 116], has been attributed to degradation of the EV membrane by exposure to bile salts upon passage through the bile duct. One may speculate that being able to change appearance during intra and inter-host transmission represents an evolutionary benefit for viruses; the membrane surrounding EV-virus allows immune evasion and genetic cooperativity inside the host but is not absolutely required for binding to or entry into host cells. At the same time, the capacity of the naked virus particles to survive and infect without a membrane allows the virus to withstand harsh conditions outside their host and to use this route for infecting new individuals. Finally, it remains to be determined whether there are more EV-editing processes that modify EV-virus composition in different tissues and contribute to their function.

## Conclusion

The conception that EV-mediated escape of naked viruses from intact cells plays a role in host-pathogen crosstalk is gradually gaining ground. Different functions have been attributed to these EV, varying from enhanced resistance against neutralizing antibodies to inducing antiviral immune responses. Being aware of and specifically addressing the heterogeneity of EV populations released by virus-infected cells can help delineating the structure-function relationship of these EV. This requires specialized knowledge and methodologies developed in the EV field to quantify and characterize different EV populations. Strong liaisons between the fields of EV biology and virology can assist in further identification of viral and/or host factors driving the formation and release of (virus-containing) EV. In the future, this may lead to the identification of new therapeutic targets to limit virus spreading and the use of virus-induced EV in body fluids as diagnostic biomarkers of viral disease.

## References

[1] Bird SW, Maynard ND, Covert MW, Kirkegaard K (2014). Nonlytic viral spread enhanced by autophagy components. Proc Natl Acad Sci U S A.

[2] Celma CCP, Roy P (2009). A viral nonstructural protein regulates bluetongue virus trafficking and release. J Virol.

[3] Jourdan N, Maurice M, Delautier D, Quero AM, Servin AL, Trugnan G (1997). Rotavirus is released from the apical surface of cultured human intestinal cells through nonconventional vesicular transport that bypasses the Golgi apparatus. J Virol.

[4] Barreto A, Rodríguez L-S, Rojas OL, Wolf M, Greenberg HB, Franco MA, Angel J (2010). Membrane vesicles released by intestinal epithelial cells infected with rotavirus inhibit T-cell function. Viral Immunol.

[5] Méndez F, Romero N, Cubas LL, Delgui LR, Rodríguez D, Rodríguez JF (2017). Non-lytic egression of infectious bursal disease virus (IBDV) particles from infected cells. PLoS One.

[6] Clayson ET, Brando LV, Compans RW (1989). Release of simian virus 40 virions from epithelial cells is polarized and occurs without cell lysis. J Virol.

[7] Bär S, Daeffler L, Rommelaere J, Nüesch JPF (2008). Vesicular egress of non-enveloped lytic parvoviruses depends on gelsolin functioning. PLoS Pathog.

[8] Altan-Bonnet N (2017). Lipid tales of viral replication and transmission. Trends Cell Biol.

[9] Chen Y-H, Du W, Hagemeijer MC (2015). Phosphatidylserine vesicles enable efficient en bloc transmission of enteroviruses. Cell.

[10] Mao L, Wu J, Shen L, Yang J, Chen J, Xu H (2016). Enterovirus 71 transmission by exosomes establishes a productive infection in human neuroblastoma cells. Virus Genes.

[11] Altan-Bonnet N (2016). Extracellular vesicles are the Trojan horses of viral infection. Curr Opin Microbiol.

[12] Raab-Traub N, Dittmer DP (2017). Viral effects on the content and function of extracellular vesicles. Nat Rev Microbiol.

[13] Yáñez-Mó M, Siljander PR-M, Andreu Z, Bedina Zavec A, Borràs FE, Buzas EI, Buzas K, Casal E, Cappello F, Carvalho J, Colás E, Cordeiro-da Silva A, Fais S, Falcon-Perez JM, Ghobrial IM, Giebel B, Gimona M, Graner M, Gursel I, Gursel M, Heegaard NHH, Hendrix A, Kierulf P, Kokubun K, Kosanovic M, Kralj-Iglic V, Krämer-Albers EM, Laitinen S, Lässer C, Lener T, Ligeti E, Linē A, Lipps G, Llorente A, Lötvall J, Manček-Keber M, Marcilla A, Mittelbrunn M, Nazarenko I, Nolte-‘t Hoen ENM, Nyman TA, O'Driscoll L, Olivan M, Oliveira C, Pállinger É, del Portillo HA, Reventós J, Rigau M, Rohde E, Sammar M, Sánchez-Madrid F, Santarém N, Schallmoser K, Stampe Ostenfeld M, Stoorvogel W, Stukelj R, van der Grein SG, Helena Vasconcelos M, Wauben MHM, de Wever O (2015). Biological properties of extracellular vesicles and their physiological functions. J Extracell Vesicles.

[14] Colombo M, Raposo G, Théry C (2014). Biogenesis, secretion, and intercellular interactions of exosomes and other extracellular vesicles. Annu Rev Cell Dev Biol.

[15] Nolte-‘t Hoen ENM, Buermans HPJ, Waasdorp M (2012). Deep sequencing of RNA from immune cell-derived vesicles uncovers the selective incorporation of small non-coding RNA biotypes with potential regulatory functions. Nucleic Acids Res.

[16] Mateescu B, Kowal EJK, van Balkom BWM, Bartel S, Bhattacharyya SN, Buzás EI, Buck AH, de Candia P, Chow FWN, Das S, Driedonks TAP, Fernández-Messina L, Haderk F, Hill AF, Jones JC, van Keuren-Jensen KR, Lai CP, Lässer C, di Liegro I, Lunavat TR, Lorenowicz MJ, Maas SLN, Mäger I, Mittelbrunn M, Momma S, Mukherjee K, Nawaz M, Pegtel DM, Pfaffl MW, Schiffelers RM, Tahara H, Théry C, Tosar JP, Wauben MHM, Witwer KW, Nolte-‘t Hoen ENM (2017). Obstacles and opportunities in the functional analysis of extracellular vesicle RNA—an ISEV position paper. J Extracell Vesicles.

[17] Trajkovic K, Hsu C, Chiantia S (2008). Ceramide triggers budding of exosome vesicles into multivesicular endosomes. Science.

[18] Tkach M, Kowal J, Zucchetti AE, Enserink L, Jouve M, Lankar D, Saitakis M, Martin-Jaular L, Théry C (2017). Qualitative differences in T-cell activation by dendritic cell-derived extracellular vesicle subtypes. EMBO J.

[19] Minciacchi VR, Spinelli C, Reis-Sobreiro M, Cavallini L, You S, Zandian M, Li X, Mishra R, Chiarugi P, Adam RM, Posadas EM, Viglietto G, Freeman MR, Cocucci E, Bhowmick NA, di Vizio D (2017). MYC mediates large oncosome-induced fibroblast reprogramming in prostate cancer. Cancer Res.

[20] Kowal J, Arras G, Colombo M, Jouve M, Morath JP, Primdal-Bengtson B, Dingli F, Loew D, Tkach M, Théry C (2016). Proteomic comparison defines novel markers to characterize heterogeneous populations of extracellular vesicle subtypes. Proc Natl Acad Sci U S A.

[21] Sun L, Wang H, Zhu X, Wu PH, Chen WQ, Zou P, Li QB, Chen ZC (2014). Serum deprivation elevates the levels of microvesicles with different size distributions and selectively enriched proteins in human myeloma cells in vitro. Acta Pharmacol Sin.

[22] Salomon C, Kobayashi M, Ashman K, Sobrevia L, Mitchell MD, Rice GE (2013). Hypoxia-induced changes in the bioactivity of cytotrophoblast-derived exosomes. PLoS One.

[23] Van Deun J, Mestdagh P, EV-TRACK Consortium J (2017). EV-TRACK: transparent reporting and centralizing knowledge in extracellular vesicle research. Nat Methods.

[24] Gould SJ, Booth AM, Hildreth JEK (2003). The Trojan exosome hypothesis. Proc Natl Acad Sci U S A.

[25] van Dongen HM, Masoumi N, Witwer KW, Pegtel DM (2016). Extracellular vesicles exploit viral entry routes for cargo delivery. Microbiol Mol Biol Rev.

[26] Nolte-‘t Hoen E, Cremer T, Gallo RC, Margolis LB (2016). Extracellular vesicles and viruses: are they close relatives?. Proc Natl Acad Sci U S A.

[27] Wurdinger T, Gatson NN, Balaj L (2012). Extracellular vesicles and their convergence with viral pathways. Adv Virol.

[28] Schwab A, Meyering SS, Lepene B, Iordanskiy S, van Hoek ML, Hakami RM, Kashanchi F (2015). Extracellular vesicles from infected cells: potential for direct pathogenesis. Front Microbiol.

[29] Feng Z, Hensley L, McKnight KL (2013). A pathogenic picornavirus acquires an envelope by hijacking cellular membranes. Nature.

[30] Robinson SM, Tsueng G, Sin J, Mangale V, Rahawi S, McIntyre LL, Williams W, Kha N, Cruz C, Hancock BM, Nguyen DP, Sayen MR, Hilton BJ, Doran KS, Segall AM, Wolkowicz R, Cornell CT, Whitton JL, Gottlieb RA, Feuer R (2014). Coxsackievirus B exits the host cell in shed microvesicles displaying autophagosomal markers. PLoS Pathog.

[31] Nagashima S, Jirintai S, Takahashi M, Kobayashi T, Tanggis, Nishizawa T, Kouki T, Yashiro T, Okamoto H (2014). Hepatitis E virus egress depends on the exosomal pathway, with secretory exosomes derived from multivesicular bodies. J Gen Virol.

[32] Sin J, McIntyre L, Stotland A, Feuer R, Gottlieb RA (2017). Coxsackievirus B escapes the infected cell in ejected mitophagosomes. J Virol.

[33] Too IHK, Yeo H, Sessions OM, Yan B, Libau EA, Howe JLC, Lim ZQ, Suku-Maran S, Ong WY, Chua KB, Wong BS, Chow VTK, Alonso S (2016). Enterovirus 71 infection of motor neuron-like NSC-34 cells undergoes a non-lytic exit pathway. Sci Rep.

[34] McKnight KL, Xie L, González-López O (2017). Protein composition of the hepatitis A virus quasi-envelope. Proc Natl Acad Sci U S A.

[35] Nagashima S, Takahashi M, Kobayashi T, Nishizawa T, Nishiyama T, Primadharsini PP, Okamoto H, Tanggis (2017). Characterization of the quasi-enveloped hepatitis E virus particles released by the cellular exosomal pathway. J Virol.

[36] Chapuy-Regaud S, Dubois M, Plisson-Chastang C, Bonnefois T, Lhomme S, Bertrand-Michel J, You B, Simoneau S, Gleizes PE, Flan B, Abravanel F, Izopet J (2017). Characterization of the lipid envelope of exosome encapsulated HEV particles protected from the immune response. Biochimie.

[37] Nagashima S, Takahashi M, Jirintai S, Tanggis, Kobayashi T, Nishizawa T, Okamoto H (2014). The membrane on the surface of hepatitis E virus particles is derived from the intracellular membrane and contains trans-Golgi network protein 2. Arch Virol.

[38] Wong J, Zhang J, Si X, Gao G, Mao I, McManus BM, Luo H (2008). Autophagosome supports coxsackievirus B3 replication in host cells. J Virol.

[39] Klein KA, Jackson WT (2011). Human rhinovirus 2 induces the autophagic pathway and replicates more efficiently in autophagic cells. J Virol.

[40] Jackson WT, Giddings TH, Taylor MP, Mulinyawe S, Rabinovitch M, Kopito RR, Kirkegaard K (2005). Subversion of cellular autophagosomal machinery by RNA viruses. PLoS Biol.

[41] O’Donnell V, Pacheco JM, LaRocco M (2011). Foot-and-mouth disease virus utilizes an autophagic pathway during viral replication. Virology.

[42] Huang S-C, Chang C-L, Wang P-S, Tsai Y, Liu HS (2009). Enterovirus 71-induced autophagy detected in vitro and in vivo promotes viral replication. J Med Virol.

[43] Reggiori F (2012). Autophagy: new questions from recent answers. ISRN Mol Biol.

[44] Kemball CC, Alirezaei M, Flynn CT, Wood MR, Harkins S, Kiosses WB, Whitton JL (2010). Coxsackievirus infection induces autophagy-like vesicles and megaphagosomes in pancreatic acinar cells in vivo. J Virol.

[45] Ponpuak M, Mandell MA, Kimura T, Chauhan S, Cleyrat C, Deretic V (2015). Secretory autophagy. Curr Opin Cell Biol.

[46] Poehler A-M, Xiang W, Spitzer P, May VEL, Meixner H, Rockenstein E, Chutna O, Outeiro TF, Winkler J, Masliah E, Klucken J (2014). Autophagy modulates SNCA/α-synuclein release, thereby generating a hostile microenvironment. Autophagy.

[47] Minakaki G, Menges S, Kittel A, Emmanouilidou E, Schaeffner I, Barkovits K, Bergmann A, Rockenstein E, Adame A, Marxreiter F, Mollenhauer B, Galasko D, Buzás EI, Schlötzer-Schrehardt U, Marcus K, Xiang W, Lie DC, Vekrellis K, Masliah E, Winkler J, Klucken J (2018). Autophagy inhibition promotes SNCA/alpha-synuclein release and transfer via extracellular vesicles with a hybrid autophagosome-exosome-like phenotype. Autophagy.

[48] Danzer KM, Kranich LR, Ruf WP, Cagsal-Getkin O, Winslow AR, Zhu L, Vanderburg CR, McLean PJ (2012). Exosomal cell-to-cell transmission of alpha synuclein oligomers. Mol Neurodegener.

[49] Alvarez-Erviti L, Seow Y, Schapira AH, Gardiner C, Sargent IL, Wood MJA, Cooper JM (2011). Lysosomal dysfunction increases exosome-mediated alpha-synuclein release and transmission. Neurobiol Dis.

[50] Vingtdeux V, Hamdane M, Loyens A, Gelé P, Drobeck H, Bégard S, Galas MC, Delacourte A, Beauvillain JC, Buée L, Sergeant N (2007). Alkalizing drugs induce accumulation of amyloid precursor protein by-products in luminal vesicles of multivesicular bodies. J Biol Chem.

[51] Simón D, García-García E, Gómez-Ramos A, Falcón-Pérez JM, Díaz-Hernández M, Hernández F, Avila J (2012). Tau overexpression results in its secretion via membrane vesicles. Neurodegener Dis.

[52] Dupont N, Jiang S, Pilli M, Ornatowski W, Bhattacharya D, Deretic V (2011). Autophagy-based unconventional secretory pathway for extracellular delivery of IL-1β. EMBO J.

[53] Zhang M, Kenny SJ, Ge L, Xu K, Schekman R (2015) Translocation of interleukin-1β into a vesicle intermediate in autophagy-mediated secretion. Elife 4:e11205. 10.7554/eLife.11205 10.7554/eLife.11205 PMC4728131 26523392

[54] Itakura E, Kishi-Itakura C, Mizushima N (2012). The hairpin-type tail-anchored SNARE syntaxin 17 targets to autophagosomes for fusion with endosomes/lysosomes. Cell.

[55] Feng Z, Hirai-Yuki A, McKnight KL, Lemon SM (2014). Naked viruses that aren’t always naked: quasi-enveloped agents of acute hepatitis. Annu Rev Virol.

[56] Nagashima S, Takahashi M, Jirintai S, Tanaka T, Nishizawa T, Yasuda J, Okamoto H (2011). Tumour susceptibility gene 101 and the vacuolar protein sorting pathway are required for the release of hepatitis E virions. J Gen Virol.

[57] Nagashima S, Takahashi M, Jirintai, Tanaka T, Yamada K, Nishizawa T, Okamoto H (2011). A PSAP motif in the ORF3 protein of hepatitis E virus is necessary for virion release from infected cells. J Gen Virol.

[58] Wirblich C, Bhattacharya B, Roy P (2006). Nonstructural protein 3 of bluetongue virus assists virus release by recruiting ESCRT-I protein Tsg101. J Virol.

[59] Witwer KW, Buzás EI, Bemis LT, Bora A, Lässer C, Lötvall J, Nolte-‘t Hoen EN, Piper MG, Sivaraman S, Skog J, Théry C, Wauben MH, Hochberg F (2013). Standardization of sample collection, isolation and analysis methods in extracellular vesicle research. J Extracell Vesicles.

[60] Taylor DD, Shah S (2015). Methods of isolating extracellular vesicles impact down-stream analyses of their cargoes. Methods.

[61] Yuana Y, Levels J, Grootemaat A, Sturk A, Nieuwland R (2014). Co-isolation of extracellular vesicles and high-density lipoproteins using density gradient ultracentrifugation. J Extracell Vesicles.

[62] Sódar BW, Kittel Á, Pálóczi K, Vukman KV, Osteikoetxea X, Szabó-Taylor K, Németh A, Sperlágh B, Baranyai T, Giricz Z, Wiener Z, Turiák L, Drahos L, Pállinger É, Vékey K, Ferdinandy P, Falus A, Buzás EI (2016). Low-density lipoprotein mimics blood plasma-derived exosomes and microvesicles during isolation and detection. Sci Rep.

[63] Heijnen HF, Schiel AE, Fijnheer R, Geuze HJ, Sixma JJ (1999). Activated platelets release two types of membrane vesicles: microvesicles by surface shedding and exosomes derived from exocytosis of multivesicular bodies and alpha-granules. Blood.

[64] Wahlund CJE, Güclüler G, Hiltbrunner S, Veerman RE, Näslund TI, Gabrielsson S (2017). Exosomes from antigen-pulsed dendritic cells induce stronger antigen-specific immune responses than microvesicles in vivo. Sci Rep.

[65] Bobrie A, Colombo M, Krumeich S, Raposo G, Théry C (2012). Diverse subpopulations of vesicles secreted by different intracellular mechanisms are present in exosome preparations obtained by differential ultracentrifugation. J Extracell Vesicles.

[66] Van Deun J, Mestdagh P, Sormunen R (2014). The impact of disparate isolation methods for extracellular vesicles on downstream RNA profiling. J Extracell Vesicles.

[67] Lobb RJ, Becker M, Wen Wen S, Wong CSF, Wiegmans AP, Leimgruber A, Möller A (2015). Optimized exosome isolation protocol for cell culture supernatant and human plasma. J Extracell Vesicles.

[68] Arraud N, Linares R, Tan S, Gounou C, Pasquet JM, Mornet S, Brisson AR (2014). Extracellular vesicles from blood plasma: determination of their morphology, size, phenotype and concentration. J Thromb Haemost.

[69] Greening DW, Xu R, Ji H (2015). A protocol for exosome isolation and characterization: evaluation of ultracentrifugation, density-gradient separation, and immunoaffinity capture methods. Methods Mol Biol.

[70] Tauro BJ, Greening DW, Mathias RA, Ji H, Mathivanan S, Scott AM, Simpson RJ (2012). Comparison of ultracentrifugation, density gradient separation, and immunoaffinity capture methods for isolating human colon cancer cell line LIM1863-derived exosomes. Methods.

[71] Tauro BJ, Greening DW, Mathias RA, Mathivanan S, Ji H, Simpson RJ (2013). Two distinct populations of exosomes are released from LIM1863 colon carcinoma cell-derived organoids. Mol Cell Proteomics.

[72] Hirai-Yuki A, Hensley L, Whitmire JK, Lemon SM (2016). Biliary secretion of quasi-enveloped human hepatitis A virus. MBio.

[73] György B, Fitzpatrick Z, Crommentuijn MHW, Mu D, Maguire CA (2014). Naturally enveloped AAV vectors for shielding neutralizing antibodies and robust gene delivery in vivo. Biomaterials.

[74] Zomer A, Maynard C, Verweij FJ, Kamermans A, Schäfer R, Beerling E, Schiffelers RM, de Wit E, Berenguer J, Ellenbroek SIJ, Wurdinger T, Pegtel DM, van Rheenen J (2015). In vivo imaging reveals extracellular vesicle-mediated phenocopying of metastatic behavior. Cell.

[75] Wiklander OPB, Nordin JZ, O’Loughlin A (2015). Extracellular vesicle in vivo biodistribution is determined by cell source, route of administration and targeting. J Extracell Vesicles.

[76] Fu Y, Zhang L, Zhang F, Tang T, Zhou Q, Feng C, Jin Y, Wu Z (2017). Exosome-mediated miR-146a transfer suppresses type I interferon response and facilitates EV71 infection. PLoS Pathog.

[77] Hoshino A, Costa-Silva B, Shen T-L, Rodrigues G, Hashimoto A, Tesic Mark M, Molina H, Kohsaka S, di Giannatale A, Ceder S, Singh S, Williams C, Soplop N, Uryu K, Pharmer L, King T, Bojmar L, Davies AE, Ararso Y, Zhang T, Zhang H, Hernandez J, Weiss JM, Dumont-Cole VD, Kramer K, Wexler LH, Narendran A, Schwartz GK, Healey JH, Sandstrom P, Jørgen Labori K, Kure EH, Grandgenett PM, Hollingsworth MA, de Sousa M, Kaur S, Jain M, Mallya K, Batra SK, Jarnagin WR, Brady MS, Fodstad O, Muller V, Pantel K, Minn AJ, Bissell MJ, Garcia BA, Kang Y, Rajasekhar VK, Ghajar CM, Matei I, Peinado H, Bromberg J, Lyden D (2015). Tumour exosome integrins determine organotropic metastasis. Nature.

[78] Mulcahy LA, Pink RC, Carter DRF (2014). Routes and mechanisms of extracellular vesicle uptake. J Extracell Vesicles.

[79] Nolte-‘t Hoen ENM, Buschow SI, Anderton SM (2009). Activated T cells recruit exosomes secreted by dendritic cells via LFA-1. Blood.

[80] Wei X, Liu C, Wang H, Wang L, Xiao F, Guo Z, Zhang H (2016). Surface phosphatidylserine is responsible for the internalization on microvesicles derived from hypoxia-induced human bone marrow mesenchymal stem cells into human endothelial cells. PLoS One.

[81] Morelli AE, Larregina AT, Shufesky WJ, Sullivan ML, Stolz DB, Papworth GD, Zahorchak AF, Logar AJ, Wang Z, Watkins SC, Falo LD, Thomson AW (2004). Endocytosis, intracellular sorting, and processing of exosomes by dendritic cells. Blood.

[82] Matsumoto A, Takahashi Y, Nishikawa M, Sano K, Morishita M, Charoenviriyakul C, Saji H, Takakura Y (2017). Role of phosphatidylserine-derived negative surface charges in the recognition and uptake of intravenously injected B16BL6-derived exosomes by macrophages. J Pharm Sci.

[83] Rimle D, Dereski W, Petty HR (1984). Enhanced binding of phosphatidylserine-containing lipid vesicle targets to RAW264 macrophages. Mol Cell Biochem.

[84] Feng Z, Li Y, McKnight KL (2015). Human pDCs preferentially sense enveloped hepatitis A virions. J Clin Invest.

[85] Das A, Hirai-Yuki A, González-López O, Rhein B, Moller-Tank S, Brouillette R, Hensley L, Misumi I, Lovell W, Cullen JM, Whitmire JK, Maury W, Lemon SM (2017). TIM1 (HAVCR1) is not essential for cellular entry of either quasi-enveloped or naked hepatitis a virions. MBio.

[86] Yin X, Ambardekar C, Lu Y, Feng Z (2016). Distinct entry mechanisms for nonenveloped and quasi-enveloped hepatitis E viruses. J Virol.

[87] Yamayoshi S, Fujii K, Koike S (2014). Receptors for enterovirus 71. Emerg Microbes Infect.

[88] Costa Verdera H, Gitz-Francois JJ, Schiffelers RM, Vader P (2017). Cellular uptake of extracellular vesicles is mediated by clathrin-independent endocytosis and macropinocytosis. J Control Release.

[89] Roberts-Dalton HD, Cocks A, Falcon-Perez JM, Sayers EJ, Webber JP, Watson P, Clayton A, Jones AT (2017). Fluorescence labelling of extracellular vesicles using a novel thiol-based strategy for quantitative analysis of cellular delivery and intracellular traffic. Nano.

[90] Tian T, Zhu Y-L, Zhou Y-Y, Liang GF, Wang YY, Hu FH, Xiao ZD (2014). Exosome uptake through clathrin-mediated endocytosis and macropinocytosis and mediating miR-21 delivery. J Biol Chem.

[91] Montecalvo A, Shufesky WJ, Stolz DB (2008). Exosomes as a short-range mechanism to spread alloantigen between dendritic cells during T cell allorecognition. J Immunol.

[92] Maguire CA, Balaj L, Sivaraman S, Crommentuijn MHW, Ericsson M, Mincheva-Nilsson L, Baranov V, Gianni D, Tannous BA, Sena-Esteves M, Breakefield XO, Skog J (2012). Microvesicle-associated AAV vector as a novel gene delivery system. Mol Ther.

[93] Takahashi M, Tanaka T, Takahashi H, Hoshino Y, Nagashima S, Jirintai, Mizuo H, Yazaki Y, Takagi T, Azuma M, Kusano E, Isoda N, Sugano K, Okamoto H (2010). Hepatitis E virus (HEV) strains in serum samples can replicate efficiently in cultured cells despite the coexistence of HEV antibodies: characterization of HEV virions in blood circulation. J Clin Microbiol.

[94] Hyenne V, Lefebvre O, Goetz JG (2017). Going live with tumor exosomes and microvesicles. Cell Adhes Migr.

[95] Guo L, Guo N (2015). Exosomes: potent regulators of tumor malignancy and potential bio-tools in clinical application. Crit Rev Oncol Hematol.

[96] Zhu X, He Z, Yuan J, Wen W, Huang X, Hu Y, Lin C, Pan J, Li R, Deng H, Liao S, Zhou R, Wu J, Li J, Li M (2015). IFITM3-containing exosome as a novel mediator for anti-viral response in dengue virus infection. Cell Microbiol.

[97] Li J, Liu K, Liu Y, Xu Y, Zhang F, Yang H, Liu J, Pan T, Chen J, Wu M, Zhou X, Yuan Z (2013). Exosomes mediate the cell-to-cell transmission of IFN-α-induced antiviral activity. Nat Immunol.

[98] Khatua AK, Taylor HE, Hildreth JEK, Popik W (2009). Exosomes packaging APOBEC3G confer human immunodeficiency virus resistance to recipient cells. J Virol.

[99] Mallegol J, van Niel G, Heyman M (2005). Phenotypic and functional characterization of intestinal epithelial exosomes. Blood Cells Mol Dis.

[100] Pallet N, Sirois I, Bell C, Hanafi LA, Hamelin K, Dieudé M, Rondeau C, Thibault P, Desjardins M, Hebert MJ (2013). A comprehensive characterization of membrane vesicles released by autophagic human endothelial cells. Proteomics.

[101] Aubertin K, Silva AKA, Luciani N, Espinosa A, Djemat A, Charue D, Gallet F, Blanc-Brude O, Wilhelm C (2016). Massive release of extracellular vesicles from cancer cells after photodynamic treatment or chemotherapy. Sci Rep.

[102] van der Vlist EJ, Nolte-‘t Hoen ENM, Stoorvogel W (2012). Fluorescent labeling of nano-sized vesicles released by cells and subsequent quantitative and qualitative analysis by high-resolution flow cytometry. Nat Protoc.

[103] Nolte-‘t Hoen ENM, van der Vlist EJ, Aalberts M (2012). Quantitative and qualitative flow cytometric analysis of nanosized cell-derived membrane vesicles. Nanomedicine.

[104] Erdbrügger U, Rudy CK, Etter ME (2014). Imaging flow cytometry elucidates limitations of microparticle analysis by conventional flow cytometry. Cytometry A.

[105] Groot Kormelink T, Arkesteijn GJA, van de Lest CHA, Geerts WJC, Goerdayal SS, Altelaar MAF, Redegeld FA, Nolte-’t Hoen ENM, Wauben MHM (2016). Mast cell degranulation is accompanied by the release of a selective subset of extracellular vesicles that contain mast cell-specific proteases. J Immunol.

[106] Segura E, Nicco C, Lombard B, Véron P, Raposo G, Batteux F, Amigorena S, Théry C (2005). ICAM-1 on exosomes from mature dendritic cells is critical for efficient naive T-cell priming. Blood.

[107] Weekes MP, Tomasec P, Huttlin EL, Fielding CA, Nusinow D, Stanton RJ, Wang ECY, Aicheler R, Murrell I, Wilkinson GWG, Lehner PJ, Gygi SP (2014). Quantitative temporal viromics: an approach to investigate host-pathogen interaction. Cell.

[108] van Hezel ME, Nieuwland R, van Bruggen R, Juffermans NP (2017). The ability of extracellular vesicles to induce a pro-inflammatory host response. Int J Mol Sci.

[109] Buzas EI, György B, Nagy G, Falus A, Gay S (2014). Emerging role of extracellular vesicles in inflammatory diseases. Nat Rev Rheumatol.

[110] Cloutier N, Tan S, Boudreau LH, Cramb C, Subbaiah R, Lahey L, Albert A, Shnayder R, Gobezie R, Nigrovic PA, Farndale RW, Robinson WH, Brisson A, Lee DM, Boilard E (2013). The exposure of autoantigens by microparticles underlies the formation of potent inflammatory components: the microparticle-associated immune complexes. EMBO Mol Med.

[111] Yuyama K, Sun H, Mitsutake S, Igarashi Y (2012). Sphingolipid-modulated exosome secretion promotes clearance of amyloid-β by microglia. J Biol Chem.

[112] Osawa S, Kurachi M, Yamamoto H, Yoshimoto Y, Ishizaki Y (2017). Fibronectin on extracellular vesicles from microvascular endothelial cells is involved in the vesicle uptake into oligodendrocyte precursor cells. Biochem Biophys Res Commun.

[113] Purushothaman A, Bandari SK, Liu J, Mobley JA, Brown EE, Sanderson RD (2016). Fibronectin on the surface of myeloma cell-derived exosomes mediates exosome-cell interactions. J Biol Chem.

[114] Bozzini S, Falcone V, Conaldi PG, Visai L, Biancone L, Dolei A, Toniolo A, Speziale P (1998). Heparin-binding domain of human fibronectin binds HIV-1 gp120/160 and reduces virus infectivity. J Med Virol.

[115] Greco G, Pal S, Pasqualini R, Schnapp LM (2002). Matrix fibronectin increases HIV stability and infectivity. J Immunol.

[116] Takahashi M, Yamada K, Hoshino Y, Takahashi H, Ichiyama K, Tanaka T, Okamoto H (2008). Monoclonal antibodies raised against the ORF3 protein of hepatitis E virus (HEV) can capture HEV particles in culture supernatant and serum but not those in feces. Arch Virol.

